# The natural history of a delayed detectable PSA after radical prostatectomy

**DOI:** 10.1038/s41391-022-00638-y

**Published:** 2023-02-10

**Authors:** Julie A. Szymaniak, Samuel L. Washington, Janet E. Cowan, Matthew R. Cooperberg, Peter E. Lonergan, Hao G. Nguyen, Maxwell V. Meng, Peter R. Carroll

**Affiliations:** 1 Department of Urology, University of California, San Francisco, CA USA; 2 Department of Epidemiology & Biostatistics, University of California, San Francisco, CA USA; 3 Department of Urology, St. James’s Hospital, Dublin, Ireland; 4 Department of Surgery, Trinity College, Dublin, Ireland

**Keywords:** Prostate cancer, Prostate cancer

## Abstract

**Introduction:**

Men with a detectable PSA after radical prostatectomy (RP) are often offered salvage therapy while those with an undetectable PSA are monitored. We aim to better characterize the natural history of men with an initially undetectable PSA who subsequently developed a detectable PSA > 6 months after RP.

**Methods:**

Retrospective analysis of men who underwent RP for clinically localized prostate cancer at the University of California, San Francisco from 2000 to 2022. The primary outcome was biochemical recurrence, defined as 2 consecutive PSA > = 0.03 ng/mL starting 6 months after surgery. Secondary outcomes were salvage treatment, post-salvage treatment, metastasis free survival (MFS), prostate cancer specific mortality (PCSM), and all-cause mortality (ACM). This cohort was compared to a previously described cohort who had an immediately detectable post-operative PSA.

**Results:**

From our cohort of 3348 patients, we identified 2868 men who had an undetectable post-op PSA. Subsequently, 642 men had a delayed detectable PSA at a median of 25 months (IQR 15, 43) with median follow-up of 72 months after RP. PSA at time of failure was <0.10 ng/mL for 65.7% of men. Of those with a delayed detectable PSA, 46% underwent salvage treatment within 10 years after RP at a median PSA of 0.08 ng/mL (IQR 0.05, 0.14). High CAPRA-S score (HR 1.09, CI 1.02–1.17, *p* = 0.02) and PSA doubling time (PSA-DT) of <6 months (HR 7.58, CI 5.42–10.6, *p* < 0.01) were associated with receiving salvage treatment. After salvage treatment, 62% of men had recurrent PSA failure within 10 years. Overall, MFS was 92%, PCSM 3%, and ACM 6% at 10 years. For those who received tertiary treatment for recurrent PSA failure, MFS was 54%, PCSM 23% and ACM 23% at 10 years’ time.

**Conclusions:**

Men who develop a detectable PSA > 6 months post-operatively may have excellent long-term outcomes, even in the absence of salvage therapy.

## Introduction

Radical prostatectomy (RP) is an effective form of definitive treatment for men with clinically localized prostate cancer. However, 20 - 40% of men may experience biochemical recurrence within 10 years [1–4]. A detectable postoperative serum PSA is thought to represent treatment failure, and some men may undergo salvage therapy [5]. However, biochemical recurrence (BCR) is increasingly recognized to have a variable course with only about a third of patients progressing to metastatic disease [6]. As such, the risks and benefits of salvage treatment must be weighed by providers and patients, in an effort to prevent clinical progression while avoiding the costs and morbidity of unnecessary treatment.

Previously, we reported on the long-term oncological outcomes of an immediately detectable (<6 months) PSA after RP, and found that the risk of development of metastasis, prostate cancer-specific mortality (PCSM), and overall survival (OM) were associated with PSA persistence [7]. In this study, we aim to better characterize the natural history of patients with a *delayed* detectable serum PSA after RP (≥6 months) using an ultrasensitive PSA cut point (0.03 ng/ml), a more contemporary value. We compare the immediate and delayed detectable PSA, and undetectable PSA groups to elucidate outcomes differences in these three distinct groups.

## Materials and methods

A retrospective analysis of men who underwent RP for non-metastatic prostate cancer at the University of California, San Francisco from 2000 to 2022 was performed utilizing the UCSF Urologic Outcomes Database after Institutional Review Board approval (#11-05329) and informed consent to participate. Exclusion criterion included cM1 disease at diagnosis. Eligible RP patients had ≥1 year of PSA follow-up data. Each participant in the delayed detectable PSA group reached an initially undetectable PSA within 6 months after RP.

The primary outcome was biochemical recurrence, defined as 2 consecutive PSA values ≥0.03 ng/mL at least 6 months after surgery. Secondary outcomes were salvage treatment, second PSA recurrence (defined as 2 consecutive PSA values ≥0.03 ng/mL), tertiary treatment, metastasis (nodal or bone) free survival (MFS), prostate cancer-specific mortality (PCSM), and all-cause mortality (ACM).

PSA results come from a variety of labs that report ultrasensitive testing. For this analysis we coded as ‘no event’ 15% of PSA results where the value was reported simply as undetectable without specifying ng/ml. Descriptive statistics were generated to describe the study cohort characteristics. Means and standard deviation (SD) or median and interquartile ranges (IQR) were reported for continuous variables, as appropriate. Counts and proportions were reported for categorical variables. Life table and Kaplan-Meier curves were used for 10-year cumulative incidence of delayed detectable PSA, salvage treatment receipt, second PSA failure, tertiary treatment, MFS, PCSM and ACM. Multivariable Cox proportional hazards regression models were utilized to identify factors associated with risk of delayed detectable PSA, salvage treatment, and second PSA recurrence. Decipher Genomic Classifier (GC) score categorized as low-, intermediate- or high-risk were based on prespecified cut-points of <0.45, 0.45–0.6, or >0.6, respectively [8]. Cancer of the Prostate Risk Assessment CAPRA post- Surgical (CAPRA-S) score categorized as low, intermediate, and high corresponded to scores of 0–2, 3–5, and ≥6, respectively [9]. Estimates were reported using hazard ratios (HR) and 95% confidence intervals (CI). Statistical analyses were performed using SAS 9.4 for Windows with *p* < 0.05 considered statistically significant.

## Results

### Cohort characteristics

**Fig. 1:**
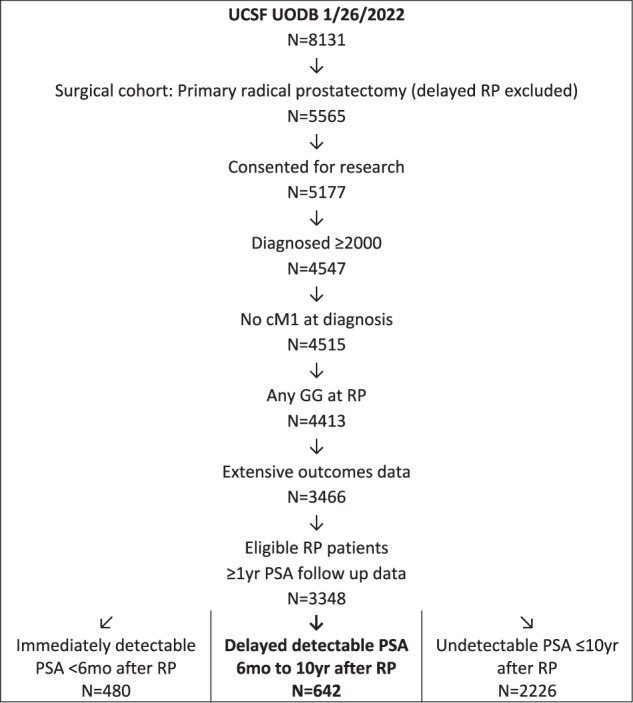
Consolidated Standards of Reporting Trials (CONSORT) diagram of study inclusion. PSA prostate‐specific antigen, UCSF University of California, San Francisco.

Of the 8131 men enrolled in the UCSF Urologic Outcomes Database as of January 2022, 5565 underwent RP and 3348 had a minimum of 1 year of follow up PSA data and had consented for enrollment. 480 men had an immediately detected (within 6 months, using the same cut–point) PSA after surgery and 2868 had an undetectable PSA post-operatively. Subsequently, 642 men progressed to a delayed detectable PSA (defined as 2 consecutive values of ≥0.03 ng/mL) while 2226 remained undetectable within 10 years [Fig. 1].

Table 1 summarizes the demographic and clinical characteristics of the entire cohort, stratified by immediate and delayed detectable PSA and undetectable PSA. After RP 642 men experienced delayed detectable PSA at a median time of 25 months (IQR 15, 53) with a median follow up of 72 months (IQR 44, 116). PSA at time of failure was <0.10 ng/mL for 65.7% of men, 0.1–0.2 ng/mL for 19%, and >0.2 ng/mL for 15.3%. The immediately detectable PSA group had the greatest proportion of men with Gleason grade group (GG) 4–5 at time of biopsy: 41% versus 24% in the delayed group and 11% in the undetectable cohort; as well as a high clinical CAPRA score (≥6) [53% versus 28% versus 12%, respectively]. Postoperatively, more patients in the immediately detectable group had pathologic GG 4-5 (37% versus 19% versus 8%), pT3/4 stage (70% versus 50% versus 25%), and high CAPRA Post-surgical (CAPRA-S) score (53% versus 20% versus 7%).

**Table 1:** Demographic and clinical characteristics of immediately and delayed detectable PSA and undetectable PSA for 3348 patients who underwent radical prostatectomy at University of California, San Francisco.

Patient Characteristics	Immediately detectable PSA < 6mo after RP *N* = 480	Delayed Detectable PSA 6mo-10yr after RP *N* = 642	Undetectable PSA within 10 yr after RP *N* = 2226	*p*-value*
**Age at diagnosis, mean (+/**−**SD)**	62.3 (6.7)	61.8 (7.4)	60.8 (7.1)	<0.01
**PSA at diagnosis, median (IQR)**	9.1 (6.2, 14.3)	6.6 (5.0, 10.0)	5.9 (4.6, 8.3)	<0.01
**Race/ethnicity,** ***n*** **(%)**				
Native American	1 (<1)	4 (1)	5 (<1)	0.15
Asian/Pacific Islander	39 (9)	41 (7)	126 (6)	
African American/Blac	20 (5)	21 (3)	65 (3)	
White	373 (86)	536 (89)	1843 (90)	
Mixed	2 (<1)	0 (0)	6 (<1)	
Missing	45	40	181	
**Biopsy Gleason grade group (GG),** ***n*** **(%)**				
GG1	56 (13)	113 (18)	897 (42)	<0.01
GG2	79 (18)	198 (32)	678 (32)	
GG3	128 (29)	158 (25)	341 (16)	
GG4/5	184 (41)	151 (24)	227 (11)	
Missing	33	22	83	
**Clinical T-stage,** ***n*** **(%)**				
T1	110 (23)	128 (20)	658 (30)	<0.01
T2	256 (54)	422 (66)	1421 (64)	
T3	107 (22)	82 (13)	131 (6)	
T4	4 (1)	7 (1)	1 (<1)	
Missing	3	3	15	
**Clinical CAPRA,** ***n*** **(%)**				
Low (0-2)	42 (11)	114 (20)	805 (42)	<0.01
Intermediate (3–5)	139 (36)	292 (52)	909 (47)	
High (>=6)	207 (53)	155 (28)	223 (12)	
Missing	92	81	289	
**Pathologic Gleason Grade group (GG),** ***n*** **(%)**				
GG1	20 (4)	47 (7)	531 (24)	<0.01
GG2	110 (23)	286 (45)	1154 (52)	
GG3	173 (36)	189 (29)	356 (16)	
GG4/5	177 (37)	120 (19)	185 (8)	
**Pathologic T-stage,** ***n*** **(%)**				
T2	145 (30)	321 (50)	1656 (74)	<0.01
T3	324 (68)	317 (49)	562 (25)	
T4	11 (2)	4 (1)	8 (0)	
**Positive margins,** ***n*** **(%)**				
No	257 (54)	459 (71)	1861 (84)	<0.01
Yes	223 (46)	183 (29)	365 (16)	
**Extracapsular extension,** ***n*** **(%)**				
No	157 (33)	338 (53)	1677 (75)	<0.01
Yes	323 (67)	304 (47)	549 (25)	
**Seminal vesicle invasion,** ***n*** **(%)**				
No	322 (67)	563 (88)	2133 (96)	<0.01
Yes	158 (33)	79 (12)	93 (4)	
**Pathologic N-stage,** ***n*** **(%)**				
NX	82 (17)	221 (35)	1247 (56)	<0.01
N0	272 (57)	391 (61)	930 (42)	
N1	126 (26)	28 (4)	36 (2)	
Missing	0	2	13	
**Decipher score at radical prostatectomy,** ***n*** **(%)**				
No test	320 (67)	481 (75)	2046 (92)	<0.01
Low < =0.45	17 (4)	44 (7)	48 (2)	
Intermediate >0.45–0.6	24 (5)	32 (5)	44 (2)	
High >0.6	119 (25)	85 (13)	88 (4)	
**Surgical CAPRA-S,** ***n*** **(%)**				
Low (0–2)	47 (10)	199 (31)	1326 (60)	<0.01
Intermediate (3–5)	178 (37)	312 (49)	750 (34)	
High (>=6)	255 (53)	131 (20)	150 (7)	

*PSA* prostate specific antigen, *CAPRA* UCSF Cancer of the Prostate Risk Assessment

*P*-value from Kruskal-Wallis test comparing median values or from Pearson chi-square test comparing categorical variables

### Predictors of a delayed detectable PSA

On multivariate Cox regression, only a high CAPRA-S score (HR 1.11, CI 1.06–1.16, *p* < 0.01) was associated with risk of delayed detectable PSA after adjustments [Table 2]. There was no association between the primary outcome and age, PSA at diagnosis, or a high Decipher score.

**Table 2:** Multivariable Cox proportional hazards regression predicting risk of delayed detectable PSA after radical prostatectomy for 3348 patients at University of California, San Francisco.

Parameter	HR (95% CI)	*p*-value
**Age at diagnosis, years**	1.01 (0.99–1.02)	0.25
**PSA at diagnosis, ng/mL**	1.01 (0.99–1.02)	0.09
**Decipher, High vs. Intermediate/Low**	1.36 (0.99–1.86)	0.06
**Surgical CAPRA-S at RP, 0–12**	1.11 (1.06–1.16)	<0.01

### Effect on long-term oncologic outcomes

The immediate versus delayed PSA groups differed in the receipt of salvage therapy (81% vs. 46%), as well as ACM (11% vs. 6%), PCSM (6% vs. 3%) and MFS (79% vs. 92%) within 10 years, respectively (Table 3). For the delayed BCR cohort, median time to metastasis was 65 months (IQR 37–92), while median time was 94 months (IQR 88, 136) for PCSM and 94 months (IQR 60, 136) for ACM.

**Table 3:** Comparison of unadjusted ten-year oncologic survival outcomes between men with an immediate versus delayed detectable PSA after radical prostatectomy for 3348 patients at University of California, San Francisco.

Outcome	DELAYED Post-operative detectable PSA*			IMMEDIATE Post-operative detectable PSA*		
	Lifetable estimate	Months to event	Still at risk at 10 yrs	Lifetable estimate	Months to event	Still at risk at 10 yrs
	% at 10 years	Median (IQR)	N	% at 10 years	Median (IQR)	*N*
Salvage treatment	46	29 (19, 46)	82	81	12 (8, 37)	17
Metastasis-free survival	92	65 (37, 92)	174	79	15 (7, 48)	47
Prostate cancer mortality	3	94 (88, 136)	174	6	89 (69, 113)	63
All-cause mortality	6	94 (60, 136)	158	11	85 (65, 144)	57

*PSA recurrence defined as 2 consecutive PSA > = 0.03 ng/ml

### Salvage Treatment

224 men (46%) with a delayed detectable PSA underwent salvage treatment within 10 years of RP. Median PSA at time of treatment was 0.08 ng/mL (IQR 0.05, 0.14) whereas the median PSA from those who did not undergo salvage therapy was 0.06 ng/mL (IQR 0.05, 0.10). Median time to treatment was 29 months (IQR 19, 46). On multivariable Cox regression, a high Decipher score (HR 2.34, CI 1.21–4.51, *p* = 0.01), a high CAPRA-S (HR 1.10, CI 1.02–1.18, *p* = 0.01) and PSA doubling time (PSA-DT) of <6 months (HR 7.58, CI 5.41–10.6, *p* < 0.01) (Table 4) were associated with risk of salvage therapy compared to those with a delayed detectable PSA who did not receive treatment. In terms of treatment type, 95.1% received radiation with or without ADT and 4.9% received ADT alone (LHRH agonist, LHRH antagonist, and/or bicalutamide). Those who received salvage therapy versus those who did not differed significantly in MFS (86% vs. 97%) and PCSM (5% vs. 1%) within 10 years, respectively (Table 5). ACM was not significantly different (7% vs. 5%, *p* = 0.53).

**Table 4:** Multivariable Cox proportional hazards regression predicting risk of salvage treatment for 3348 patients who underwent radical prostatectomy at University of California, San Francisco.

Parameter	HR (95% CI)	*p* value
**Age at diagnosis, years**	0.99 (0.97–1.00)	0.08
**PSA at diagnosis, ng/ml**	1.01 (0.99–1.03)	0.13
**Decipher, High vs. Intermediate/Low**	2.34 (1.21–4.51)	0.01
**Surgical CAPRA-S at RP, 0-12**	1.10 (1.02–1.18)	0.01
**PSA-DT <6 months (Yes vs. No)**	7.58 (5.42–10.6)	<0.01

**Table 5:** Comparison of unadjusted ten-year oncologic survival outcomes between men with a delayed detectable PSA for 3348 patients who underwent radical prostatectomy at University of California, San Francisco.

Outcome	Salvage Treatment		
	Yes	No	*p*-value
Metastasis-free survival	86%	97%	<0.01
Prostate cancer-specific mortality	5%	1%	0.05
All-cause mortality	7%	5%	0.53

### Outcomes after salvage treatment

After salvage treatment, 62% of men had recurrent PSA failure within 10 years, at a median time of 34 months (IQR 19-56). PSA-DT was <6 months for 85.2% of these patients. On multivariable Cox regression, only PSA-DT < 6 months was associated with risk of recurrence following salvage therapy (HR 6.52, CI 3.51–12.14, *p* < 0.01). There was no association between PSA at diagnosis, Decipher score, or CAPRA-S and the likelihood of failing salvage treatment (Table 6).

**Table 6:** Multivariable Cox proportional hazards regression predicting risk of second PSA recurrence after salvage treatment for 3348 patients who underwent radical prostatectomy at University of California, San Francisco.

Parameter	HR (95% CI)	*p*-value
**Age at diagnosis, years**	1.02 (0.99–1.06)	0.27
**PSA at diagnosis, ng/ml**	0.99 (0.97–1.02)	0.87
**Decipher, High vs. Intermediate/Low**	0.79 (0.20–3.15)	0.74
**Surgical CAPRA-S at RP, 0-12**	1.06 (0.92–1.23)	0.4
**PSA-DT <6 months after salvage tx, Yes vs. No**	6.52 (3.51–12.14)	<0.01

Those who received tertiary treatment versus those who were monitored differed significantly in ACM (23% vs. 3%), PCSM (23% vs. 0%) and MFS (54% vs. 91%), respectively (Table 7). In terms of tertiary salvage treatment type, 12.8% underwent radiation, 78.7% received ADT alone, and 8.5% had advanced systemic treatment, which included enzalutamide, abiraterone, ketoconazole, chemotherapy agents, and/or immunotherapies.

**Table 7:** Comparison of unadjusted ten-year oncologic survival outcomes between men with a detectable post-salvage treatment PSA for 3348 patients who underwent radical prostatectomy at University of California, San Francisco.

Outcome	Tertiary treatment		No tertiary treatment		
	Lifetable estimate	Still at risk at 10 yrs	Lifetable estimate	Still at risk at 10 yrs	*p*-value
	% at 10 years	*N*	% at 10 years	*N*	
Metastasis-free survival	54	10	91	57	<0.01
Prostate cancer mortality	23	14	0	60	<0.01
All-cause mortality	23	14	3	60	<0.01

*All log-rank *p* < 0.01

## Discussion

In this study of 3348 men treated with RP for clinically localized prostate cancer, we found that 14.3% of patients had immediate PSA persistence while 19.1% developed biochemical recurrence ≥6 months within 10 years of primary treatment. Median time to BCR in the delayed detectable PSA group was 25 months (IQR 15-43), and PSA at time of failure was <0.10 ng/mL for 65.7% of patients. Increasing CAPRA-S score was a risk factor associated with development of delayed detectable PSA. CAPRA-S score is computed from preoperative PSA, pathologic Gleason score, extracapsular extension, lymph node involvement, positive surgical margin, and seminal vesicle invasion [9]. Previous studies have similarly found that seminal vesicle invasion, pathologic Gleason score, and positive surgical margin are significant prognostic factors for biochemical recurrence [6, 10, 11]. As such, we included CAPRA-S in our analysis rather than its component parts. Overall, long-term oncologic outcomes were excellent for the delayed detectable PSA group, with MFS 92%, PCSM 3%, and ACM 6% at 10 years. PCSM is similar to previously published outcomes [12].

Of those with a delayed detectable PSA, 46% underwent salvage treatment within 10 years after RP. This is higher than our previously published CaPSURE cohort treatment rate of 34% for BCR [5]. Several differences between the cohorts may explain this finding: the CaPSURE study included patients treated between 1995-2002 with BCR defined as PSA ≥ 0.2 ng/mL whereas this study involves a more contemporary group with a lower PSA cut-off of 0.03 ng/mL. The majority of those patients had biopsy Gleason score 2-6 (66.8%) versus 18% in this study. Furthermore, median follow up in the CaPSURE group was shorter, at 63.5 months compared to 72 months.

The median PSA of patients undergoing salvage therapy was 0.08 ng/mL (IQR 0.05, 0.14) and median time to treatment was 29 months (IQR 19, 46). Though the PSA level at time of salvage therapy in our cohort is lower than the PSA trigger level of 0.2 ng/mL in the RAVES and GETUG-AFU 17 randomized trials, our findings similarly support salvage treatment in appropriately selected patients, with favorable long-term oncologic outcomes of MFS 86% and PCSM 5% [13].

Most patients who received salvage treatment underwent radiation with or without ADT (95.1%), while the remainder were placed on ADT alone. We found that a high Decipher score (HR 2.34, CI 1.21–4.51, *p* = 0.1), an increasing CAPRA-S score (HR 1.09, CI 1.02-1.17, *p* = 0.02) and PSA-DT of <6 months (HR 7.58, CI 5.42-10.6, *p* < 0.01) were associated with risk of receiving salvage treatment. In a review on management of BCR after primary treatment, Artibani et al reported that a PDA-DT of <3 months was associated with high risk of metastases and PCSM [3].

Shahait et al recently analyzed the use of post-prostatectomy Decipher risk stratification in two prospective observational cohorts and found that those with a high Decipher score were more likely to receive secondary therapy compared to those with low/intermediate Decipher risk, with OR 6.84 [14]. We also found that a high Decipher score was associated with risk of salvage treatment (HR 2.34), but not with recurrent PSA failure.

After salvage treatment, 62% of men had recurrent PSA failure within 10 years, at a median of 34 months. PSA-DT < 6 months after salvage therapy was associated with risk of second PSA failure. Of those receiving tertiary treatment, 78.7% had ADT alone while 8.5% received systemic therapy, including enzalutamide, abiraterone, ketoconazole, chemotherapy, and immunotherapies. Tumati et al reported on a smaller cohort of 286 patients who underwent salvage RT between 1986 and 2013 and subsequently developed second BCR. At a median of 6.1 years after second BCR, metastasis, PCSM and ACM were 41%, 17.7% and 27.1%, respectively [15]. Similarly, for those who received tertiary treatment for recurrent PSA failure in our cohort, we found MFS was 54%, PCSM 23% and ACM 23% at 10 years’ time. Remarkably, some patients who progress through salvage and tertiary therapy may expect a prolonged clinical course rather than a rapid decline.

Our study has several strengths, including a more contemporary definition of detectable PSA as ≥0.03 ng/ml and a median follow-up period of 6 years for the delayed BCR cohort. Additionally, our database includes patients who had a second BCR after salvage treatment as well as data on tertiary treatments. Limitations include the retrospective nature of this study within a single-center experience of patients who underwent surgery. As UCSF is a tertiary referral center, it is likely that patients with a detectable PSA will continue their care at our institution while those without recurrence may be at higher risk of loss to follow-up at UCSF. Additionally, the clinical generalizability of our findings is limited by our institutional use of Decipher and CAPRA scores, which may not be widely adopted in the community setting. Furthermore, detailed information on how decisions for salvage and tertiary treatment were made are lacking in this natural history study.

## Conclusion

Men who develop a detectable serum PSA > 6 months post-operatively may have excellent long-term outcomes. A high Decipher score, increasing CAPRA-S, and short PSA doubling-time are associated with risk of receiving salvage treatment. Such information can be used to assess the timing and type of additional treatment for patients with biochemical recurrence.

## Data Availability

The research data for this study are stored on servers servicing the University of California Urologic Outcomes Database. The data can be accessed by contacting the corresponding author.
